# Short term synbiotic supplementation improves hepatic and metabolic outcomes in children with metabolic dysfunction associated steatotic liver disease

**DOI:** 10.1038/s41598-026-54652-4

**Published:** 2026-05-25

**Authors:** Maryam Naseri Taheri, Abbas Taghaviardakani, Reza Hosseiniara, Javid Azadbakht, Mohammad Reza Sharif, Davood Kheirkhah

**Affiliations:** 1 Department of Pediatrics, Kashan University of Medical Sciences, Kashan, Iran; 2 Department of Pediatrics, Infectious Diseases Research Center, School of Medicine, Shahid Beheshti Hospital, Kashan University of Medical Sciences, Kashan, Iran; 3 Research Center for Biochemistry and Nutrition in Metabolic Diseases, Kashan University of Medical Sciences, Kashan, Iran; 4 Department of Radiology, Faculty of Medicine, Kashan University of Medical Sciences, Kashan, Iran; 5 Department of Pediatrics, School of Medicine, Shahid Beheshti Hospital, Kashan University of Medical Sciences, Kashan, Iran

**Keywords:** Metabolic dysfunction-associated steatotic liver disease, Pediatrics, Synbiotics, Lipid profile, Liver enzyme activities

## Abstract

Metabolic dysfunction-associated steatotic liver disease (MASLD) is an increasingly recognized chronic liver disorder in children and adolescents, particularly among those with obesity and insulin resistance. Given the emerging role of the gut–liver axis in disease pathogenesis, this study aimed to evaluate the effectiveness of synbiotic supplementation in improving biochemical and ultrasonographic outcomes in pediatric MASLD. This double-blind, randomized, placebo-controlled clinical trial was conducted over 8 weeks. A total of 60 participants aged 10–18 years with MASLD were randomly assigned to receive either synbiotics (*n* = 30) or placebo (*n* = 30). The synbiotic formulation contained a combination of nine probiotic strains with a prebiotic component, whereas the placebo consisted of prebiotic-only capsules (fructooligosaccharide with sucrose). The primary outcomes were predefined as changes in liver enzyme activities (alanine aminotransferase [ALT] and aspartate aminotransferase [AST]) and hepatic steatosis grade assessed by ultrasonography, while secondary outcomes included body mass index (BMI) and lipid profile parameters. Between-group differences were analyzed using appropriate statistical models adjusting for baseline values. After 8 weeks, the synbiotics group showed statistically significant reductions in BMI (− 0.70 ± 0.16 kg/m^2^), triglycerides (− 10.50 ± 3.10 mg/dL), total cholesterol (− 10.50 ± 2.90 mg/dL), and LDL-cholesterol (− 5.46 ± 1.85 mg/dL) compared with the placebo group (all *p* < 0.001). Liver enzyme activities decreased significantly in the synbiotics group (ALT − 20.36 ± 7.18 U/L; AST − 16.76 ± 10.67 U/L), with greater reductions than in the placebo group (*p* < 0.001 for between-group comparisons). Furthermore, improvement in hepatic steatosis grade was observed in 73.3% of participants in the synbiotics group compared to 23.3% in the placebo group (*p* < 0.001). Synbiotic supplementation over an 8-week period was associated with significant improvements in liver enzyme activities, lipid profile, BMI, and ultrasonographic steatosis grade in children with MASLD. While these findings suggest a beneficial role for synbiotics as an adjunctive strategy, the relatively short intervention period and lack of control for lifestyle factors warrant cautious interpretation. Further studies with longer follow-up, detailed lifestyle assessment, and mechanistic investigations are needed to confirm these results and clarify the underlying pathways.

*Iranian Registry of Clinical Trials code*: IRCT20220314054279N1.

## Introduction

Metabolic dysfunction-associated steatotic liver disease (MASLD) is a chronic and increasingly prevalent liver disorder characterized by the accumulation of fat in hepatocytes in the absence of significant alcohol consumption. The disease encompasses a broad histopathological spectrum, ranging from simple steatosis to metabolic dysfunction-associated steatohepatitis (MASH), which is defined by hepatocellular injury, inflammation, and varying degrees of fibrosis, and may ultimately progress to cirrhosis or hepatocellular carcinoma^1^. In recent years, MASLD has emerged as the most common cause of chronic liver disease in the pediatric population, paralleling the global rise in childhood obesity and metabolic dysfunction. Contemporary epidemiological data indicate not only an increasing global prevalence but also important demographic variations, including higher rates in boys, a disproportionate burden among Hispanic populations, and a strong association with insulin resistance and central adiposity. Current estimates suggest that MASLD affects approximately 10–20% of children worldwide, with substantially higher prevalence among those with obesity or features of metabolic syndrome^2,3^.

In pediatric populations, MASLD is closely linked to metabolic syndrome, encompassing visceral obesity, insulin resistance, dyslipidemia, and impaired glucose metabolism. The pathogenesis of MASLD is multifactorial and involves a complex interplay between genetic susceptibility, environmental exposures, and metabolic disturbances. Dietary patterns characterized by high caloric intake, sedentary behavior, and altered energy homeostasis play a central role in disease development^4^. A key mechanistic pathway is the gut–liver axis, whereby alterations in gut microbiota composition -referred to as dysbiosis- contribute to increased intestinal permeability, endotoxemia, and activation of inflammatory signaling pathways. Translocation of bacterial products such as lipopolysaccharides into the portal circulation can activate toll-like receptors, thereby promoting hepatic inflammation, insulin resistance, and lipid dysregulation^5^.

Current management strategies for MASLD primarily rely on lifestyle modification, including dietary changes, weight reduction, and increased physical activity. Despite their central role, these interventions are often difficult to implement and sustain in pediatric populations, resulting in variable adherence and limited long-term effectiveness. Although several pharmacological agents, including insulin sensitizers and antioxidants, have shown some benefit, no therapies are currently approved specifically for pediatric MASLD or MASH, emphasizing the need for alternative and adjunctive treatment strategies^6,7^.

Increasing attention has been directed toward the therapeutic modulation of the gut microbiota, given its pivotal role in the pathophysiology of MASLD. The gut-liver axis represents a bidirectional communication network in which microbial metabolites, immune signaling, and intestinal barrier integrity collectively influence hepatic function^8–10^. Synbiotics, defined as combinations of probiotics and prebiotics designed to synergistically enhance microbial balance, have emerged as a promising intervention. These agents may improve intestinal barrier integrity, reduce endotoxin translocation, and modulate systemic and hepatic inflammatory responses^11^.

Clinical evidence suggests that probiotics and synbiotics can favorably influence several metabolic and hepatic parameters relevant to MASLD. Proposed mechanisms include the production of short-chain fatty acids (SCFAs), modulation of bile acid metabolism, and attenuation of inflammatory pathways^12,13^. Certain probiotic strains have been associated with reductions in liver enzyme activities (e.g., alanine aminotransferase [ALT] and aspartate aminotransferase [AST]), improvements in lipid profiles, and decreased hepatic fat accumulation^12^. Meta-analyses of randomized controlled trials have reported improvements in liver enzyme activities and inflammatory markers; however, these findings are largely derived from adult populations^14^. Consequently, the applicability of these results to children remains uncertain, particularly given developmental differences in metabolism, microbiome composition, and disease progression.

Preliminary pediatric studies suggest that synbiotics may offer clinical benefits in children with MASLD. However, the available evidence remains limited and heterogeneous, with substantial variability in probiotic strains, dosages, treatment duration, and outcome measures^15–17^. Moreover, few studies have explored mechanistic endpoints, such as microbiome composition or metabolite profiles, which limits the ability to establish causal pathways underlying observed clinical effects.

The diagnosis and assessment of MASLD in children also presents distinct challenges. While elevated liver enzyme activities and ultrasonography are commonly used in clinical practice, these approaches have important limitations, including low sensitivity for mild steatosis and the inability to accurately stage fibrosis. Liver biopsy remains the reference standard for diagnosing MASH and assessing disease severity; however, its invasive nature restricts routine use in pediatric populations. Additionally, pediatric-specific considerations -such as sex-specific alanine aminotransferase thresholds and validated scoring systems for disease severity- are critical for accurate evaluation but are not consistently applied^18,19^.

Given these challenges, there is a clear need for well-designed clinical trials that evaluate both the efficacy and limitations of emerging therapies while accounting for key confounding factors such as diet and physical activity. Furthermore, the relatively short duration of most existing studies raises important questions regarding the sustainability and clinical relevance of observed improvements in this chronic condition.

Accordingly, the present randomized, double-blind, placebo-controlled trial was designed to evaluate the effects of synbiotic supplementation on liver enzyme activities, lipid profile, and ultrasonographic features in children and adolescents with MASLD.

## Methods

### Study design

This study was designed as a randomized, double-blind, placebo-controlled clinical trial to evaluate the effects of synbiotic supplementation on liver enzyme activities, lipid profile, and ultrasonographic features in children and adolescents aged 10–18 years diagnosed with metabolic dysfunction-associated steatotic liver disease (MASLD). The intervention period was limited to 8 weeks based on prior pediatric studies^17,20,21^ demonstrating short-term metabolic responsiveness to probiotic supplementation; however, this duration was selected as an initial exploratory timeframe rather than to assess long-term efficacy.

### Participants

We recruited 60 children with MASLD from outpatient clinics in Kashan, Iran. Inclusion criteria required participants to be aged between 10 and 18 years, with persistently elevated alanine aminotransferase (ALT) levels (> 40 U/L) confirmed on two separate occasions over a one-month interval, and ultrasonographic evidence of hepatic steatosis.

Given the absence of universally accepted pediatric diagnostic criteria, ALT thresholds were applied pragmatically; however, it is acknowledged that sex-specific pediatric cutoffs may provide greater diagnostic accuracy. Anthropometric measurements, including height, weight, and BMI, were assessed at baseline and at the end of the study period using standardized protocols. BMI percentiles were calculated based on age and sex. Although overweight or obesity were not mandatory inclusion criteria, baseline BMI distribution was comparable between groups. Waist circumference, an important indicator of visceral adiposity, was not measured and is recognized as a limitation.

Exclusion criteria were a history of alcohol consumption, systemic diseases such as diabetes, inflammatory or autoimmune disorders, hypothyroidism, or viral hepatitis, and a clinical suspicion of metabolic dysfunction-associated steatohepatitis (MASH). Due to ethical and practical considerations in pediatric populations, liver biopsy -the reference standard for diagnosing MASH and staging fibrosis-was not performed; instead, exclusion was based on clinical, biochemical, and ultrasonographic findings, which may limit diagnostic precision. Participants who had used antibiotics or probiotics within three months prior to enrollment were excluded. Medication history and comorbid conditions were assessed through clinical records and parental reporting. Other causes of steatotic liver, including Wilson disease and celiac disease, were excluded based on clinical evaluation and appropriate laboratory testing when indicated.

Data on dietary intake, physical activity, and lifestyle behaviors were not systematically collected during the study period; participants were advised to maintain their usual habits. This lack of detailed lifestyle assessment is acknowledged as a potential confounding factor.

### Randomization and blinding

Participants were randomly assigned to either the synbiotics group (*n* = 30) or the placebo group (*n* = 30) using a block randomization method with a 1:1 allocation ratio. The randomization sequence was generated using an online tool (Sealed Envelope, www.sealedenvelope.com) and was concealed until assignment. Allocation concealment was ensured using sequentially numbered containers. Both participants and research staff, including outcome assessors and statisticians, remained blinded to group allocation throughout the study.

### Sample size calculation

Sample size estimation was based on a previous study by Famouri et al.^20^ which reported improvement in ultrasonographic outcomes in 53.1% of the intervention group compared to 16.5% in controls. Accordingly, the primary outcome for sample size estimation was defined as improvement in hepatic steatosis grade assessed by ultrasonography. To achieve 90% power with a two-sided alpha of 0.05, a minimum of 30 participants per group was required.

Although liver enzyme activities were also key outcomes, the study was not specifically powered to detect smaller between-group differences in biochemical parameters.

### Interventions

The synbiotics group received capsules containing 500 mg of probiotics (Familact®), comprising the following strains:

- *Lactobacillus rhamnosus*
- *L. casei*
- *L. acidophilus*
- *L. plantarum*
- *Bifidobacterium lactis*
- *B. breve*
- *B. longum*
- *B. bifidum*
- *Streptococcus thermophilus*

The total viable count was reported by the manufacturer; however, strain-specific colony-forming unit (CFU) distribution was not available, which may limit reproducibility and comparison with other studies. The formulation also contained 21 mg of fructooligosaccharide (FOS) as a prebiotic component.

The placebo group received identical capsules containing only FOS (21 mg) with sucrose and no live microorganisms. Given the known prebiotic activity of FOS, its inclusion in the placebo may have exerted mild biological effects and potentially attenuated the observed between-group differences; this was considered in the interpretation of results.

Participants were instructed to take one capsule daily after meals for 8 weeks. The selected dose and formulation were based on prior pediatric studies^15,20,22^ demonstrating safety and potential efficacy of multi-strain synbiotics in MASLD. Storage conditions were explained to ensure product stability.

### Adherence monitoring and safety

Participants were contacted weekly by telephone to monitor adherence and address any concerns. They were asked to return empty supplement packages at follow-up visits. Three visits were scheduled: baseline, week 4, and week 8. Adherence was considered acceptable if ≥ 80% of capsules were consumed. No adverse events or gastrointestinal complications were reported.

### Outcome measures

Assessments were conducted at baseline and after the 8-week intervention:

- **Anthropometric measurements:** BMI was calculated using standardized procedures.
- **Laboratory tests:** Fasting blood samples were analyzed for fasting blood sugar (FBS), lipid profile (triglycerides, total cholesterol, HDL-C, LDL-C), and liver enzyme activities (ALT and AST).
- **Sonographic assessment:** Liver ultrasound was performed using a WS80 scanner by a blinded radiologist. Although ultrasound is widely used in clinical practice, it has limited sensitivity for detecting mild steatosis and cannot reliably assess fibrosis severity. Fatty liver grading was defined as:
  o Grade I: Mild increase in echogenicity
  o Grade II: Moderate increase
  o Grade III: Severe increase

### Statistical analysis

Data were analyzed using SPSS (version 16). Continuous variables were expressed as mean ± SD, and categorical variables as frequency (percentage).

Normality of data distribution was assessed using the Shapiro–Wilk test. Within-group comparisons were performed using paired t-tests for normally distributed variables. Between-group comparisons were conducted using analysis of covariance (ANCOVA), adjusting for baseline values. Independent t-tests were used for change scores in liver enzyme activities due to violation of ANCOVA assumptions.

In addition to absolute changes, percentage changes from baseline were calculated to facilitate clinical interpretation. Differences in fatty liver grades were analyzed using ordinal logistic regression. A p-value < 0.05 was considered statistically significant. All analyses were performed by a blinded statistician.

### Ethical considerations

The study was approved by the Research Ethics Committee of Kashan University of Medical Sciences (IR.KAUMS.MEDNT.REC.1399.144) and registered in the Iranian Registry of Clinical Trials (IRCT20220314054279N1). Written informed consent was obtained from parents or guardians. The study adhered to the principles of the Declaration of Helsinki.

## Results

### Demographics and baseline characteristics

**Fig. 1:**
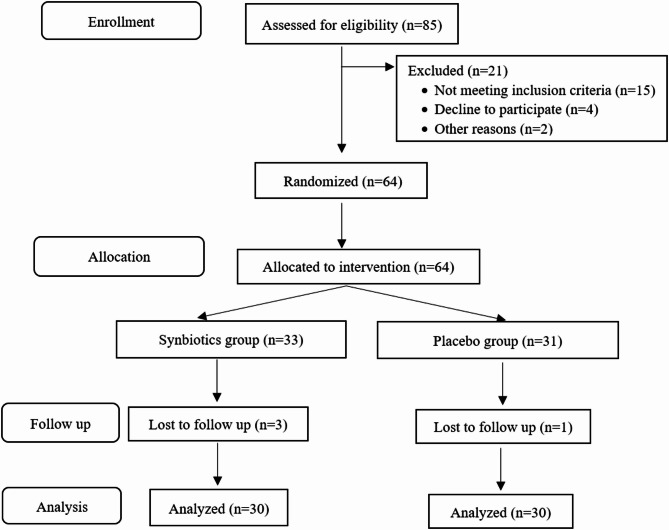
Consort flowchart for participants selection.

A total of 60 children diagnosed with MASLD completed the study (Fig. 1), with participants evenly distributed between the synbiotics group (*n* = 30; 17 boys, mean age 13.37 ± 1.83 years) and the placebo group (*n* = 30; 16 boys, mean age 13.83 ± 2.52 years). No statistically significant differences were observed between groups at baseline with respect to age, sex distribution, BMI, or biochemical parameters (all *p* > 0.05), indicating appropriate comparability prior to intervention.

### BMI, fasting blood sugar, and lipid profiles

Table 1 presents the comparison of BMI, fasting blood sugar (FBS), and lipid profiles between the synbiotics and placebo groups at baseline and after 8 weeks. Between-group analysis using ANCOVA (adjusted for baseline values) demonstrated statistically significant reductions in BMI, triglycerides, total cholesterol, and LDL-C in the synbiotics group compared with the placebo group (all *p* < 0.001).

**Table 1:** Comparison of BMI, fasting blood sugar, and lipid profiles between groups.

Variable	Synbiotics			Placebo			*p*-value† (Betwee*n*-group)
	Baseline	8 weeks	*p*-value‡	Baseline	8 weeks	*p*-value‡	
BMI (kg/m^2^)	24.04 ± 1.87	23.34 ± 1.71	< 0.001	23.67 ± 3.08	23.52 ± 3.00	0.120	< 0.001
FBS (mg/dL)	92.23 ± 9.11	91.07 ± 7.02	0.205	91.67 ± 9.58	90.17 ± 6.68	0.192	0.595
Triglycerides (mg/dL)	121.50 ± 30.61	111.00 ± 27.51	< 0.001	118.73 ± 24.65	115.23 ± 22.15	0.011	< 0.001
Total Cholesterol (mg/dL)	157.00 ± 18.45	146.50 ± 18.18	< 0.001	155.70 ± 15.31	153.37 ± 15.52	0.011	< 0.001
HDL-C (mg/dL)	40.83 ± 4.71	41.60 ± 4.91	0.062	41.47 ± 6.61	42.03 ± 5.81	0.134	0.816
LDL-C (mg/dL)	92.13 ± 13.67	86.67 ± 14.40	< 0.001	90.73 ± 9.91	89.73 ± 10.18	0.044	< 0.001

Values are presented as mean ± standard deviation (SD). †Between-group comparisons were performed using analysis of covariance (ANCOVA), adjusted for baseline values. ‡Within-group comparisons were performed using paired t-tests. FBS, fasting blood sugar; HDL-C, high-density lipoprotein cholesterol; LDL-C, low-density lipoprotein cholesterol.

Within-group analyses showed that the synbiotics group experienced significant reductions in BMI (− 0.70 ± 0.16 kg/m^2^; − 2.9%), triglycerides (− 10.50 ± 3.10 mg/dL; − 8.6%), total cholesterol (− 10.50 ± 2.90 mg/dL; − 6.7%), and LDL-C (− 5.46 ± 1.85 mg/dL; − 5.9%) over the 8-week period (all *p* < 0.001).

In contrast, the placebo group demonstrated no significant change in BMI (− 0.15 ± 0.08 kg/m^2^; *p* = 0.120) and only modest reductions in triglycerides (− 3.50 ± 2.50 mg/dL; *p* = 0.011) and LDL-C (− 1.00 ± 0.90 mg/dL; *p* = 0.044), with no clinically meaningful changes in total cholesterol or HDL-C. FBS and HDL-C remained unchanged in both groups, with no statistically significant between-group differences (*p* > 0.05).

### Liver enzyme activities

Table 2 summarizes the liver enzyme data. Between-group comparisons of change scores revealed significantly greater reductions in liver enzyme activities in the synbiotics group compared with the placebo group for both AST and ALT (*p* < 0.001).

**Table 2:** Comparison of liver enzyme activities between groups.

Variable	Synbiotics				Placebo				*p*-value† (Between-group)
	Baseline	8 weeks	*p*-value‡	Change (Δ)	Baseline	8 weeks	*p*-value‡	Change (Δ)	
AST (U/L)	74.73 ± 33.71	57.97 ± 25.07	< 0.001	− 16.76 ± 10.67	68.77 ± 24.20	61.67 ± 21.84	< 0.001	− 7.10 ± 4.13	< 0.001
ALT (U/L)	82.33 ± 29.61	61.97 ± 22.48	< 0.001	− 20.36 ± 7.18	81.30 ± 18.69	73.50 ± 16.65	< 0.001	− 7.80 ± 2.28	< 0.001

Values are presented as mean ± SD. † Between-group comparisons were performed using independent t-tests on change scores (Δ = post − pre). ‡ Within-group comparisons were performed using paired t-tests. AST: Aspartate Aminotransferase; ALT: Alanine Aminotransferase.

In the synbiotics group, AST decreased by − 16.76 ± 10.67 U/L (− 22.4%), and ALT decreased by − 20.36 ± 7.18 U/L (− 24.7%), both of which were statistically significant (*p* < 0.001). In contrast, the placebo group also showed reductions in AST (− 7.10 ± 4.13 U/L; − 10.3%) and ALT (− 7.80 ± 2.28 U/L; − 9.6%), but these changes were significantly smaller in magnitude.

### Fatty liver grade improvement

Ordinal logistic regression analysis (Table 3) demonstrated a statistically significant difference in the distribution of fatty liver grades between the two groups (*p* < 0.001). After 8 weeks, 73.3% of participants in the synbiotics group showed improvement in hepatic steatosis grade, compared with 23.3% in the placebo group, corresponding to an absolute difference of 50%.

**Table 3:** Comparison of fatty liver grade between groups.

Fatty liver grade	Synbiotics			Placebo			*p*-value† (Between-group)
	Baseline	8 weeks	*p*-value‡	Baseline	8 weeks	*p*-value‡	
Normal	0	16 (53.3%)		0	4 (13.3%)		
Grade I	16 (53.3%)	9 (30.0%)		18 (60.0%)	17 (56.7%)		
Grade II	14 (46.7%)	5 (16.7%)	< 0.001	12 (40.0%)	9 (30.0%)	0.003	< 0.001

Values are presented as frequency (percentage). †Between-group differences were analyzed using ordinal logistic regression. ‡Within-group changes were assessed using ordinal regression models. Improvement defined as a reduction in steatosis grade from baseline.

Specifically, in the synbiotics group, 13 participants improved from grade I to normal, three from grade II to normal, and six from grade II to grade I. In contrast, the placebo group showed more limited improvement, with four participants transitioning from grade I to normal and three from grade II to grade I.

## Discussion

The results of this randomized, double-blind, placebo-controlled clinical trial demonstrate that synbiotic supplementation is associated with significant short-term improvements in metabolic and hepatic parameters in children with MASLD; however, these findings should be interpreted with appropriate caution given methodological and contextual limitations. Over an 8-week period, statistically significant between-group differences were observed in BMI, lipid profiles, liver enzyme activities, and ultrasonographic grading of hepatic steatosis. While these results support a potential therapeutic role for synbiotics, the magnitude and durability of these effects require further investigation in longer-term studies.

The observed reduction in BMI in the synbiotics group suggests that modulation of the gut microbiota may influence weight regulation in pediatric MASLD. This finding is consistent with prior pediatric studies reporting modest reductions in BMI following probiotic supplementation^15,17^. Notably, the magnitude of BMI reduction in the present study appears greater than that reported in some earlier trials, which may reflect differences in strain composition, synbiotic formulation, or baseline metabolic characteristics of the study population. Mechanistically, synbiotics may influence host energy balance through modulation of microbial composition, regulation of appetite-related hormones, and attenuation of low-grade systemic inflammation^23,24^. However, it is important to note that dietary intake and physical activity were not systematically assessed in this study, and therefore, residual confounding by lifestyle factors cannot be excluded. This limitation complicates the attribution of weight changes solely to synbiotic supplementation.

Improvements in lipid parameters, particularly reductions in triglycerides, total cholesterol, and LDL-C, are in line with previous findings^15,20^. These effects may be mediated through alterations in bile acid metabolism, decreased intestinal cholesterol absorption, and enhanced microbial production of metabolites that regulate lipid homeostasis^25,26^. However, the absence of direct measurements of bile acids or microbiome composition in the present study limits the ability to confirm these mechanistic pathways. Additionally, the relatively modest lipid changes observed in the placebo group may reflect the biological activity of fructooligosaccharide (FOS), which was included as a prebiotic component in the placebo formulation and could have partially attenuated between-group differences.

In contrast to lipid outcomes, no significant changes were observed in fasting blood sugar or HDL-C levels. This finding is consistent with some previous reports^20,27^, but contrasts with others, highlighting the heterogeneity of metabolic responses to probiotic interventions. The lack of glycemic improvement may be attributable to the relatively short intervention period, as well as potential strain-specific effects that were not fully characterized in this study. Indeed, longer intervention durations and targeted probiotic formulations may be necessary to achieve clinically meaningful improvements in glucose metabolism^21^.

A key finding of this study is the significant reduction in liver enzyme activities, including ALT and AST, in the synbiotics group. These results are consistent with meta-analytic evidence supporting the hepatoprotective effects of microbiome-modulating therapies^27,28^. However, while reductions in liver enzyme activities are commonly interpreted as indicators of improved hepatic status, they do not necessarily reflect histological improvement, particularly in the absence of liver biopsy data^29,30^. Therefore, the clinical significance of these biochemical changes should be interpreted cautiously.

The improvement in ultrasonographic grading of hepatic steatosis represents one of the most clinically relevant findings of this study. The proportion of patients demonstrating improvement (73.3%) was substantially higher in the synbiotics group compared with the placebo group (23.3%), indicating a meaningful treatment effect. Similar findings have been reported in pediatric and adult populations^15,22^. Nevertheless, ultrasonography has inherent limitations, including reduced sensitivity for mild steatosis and inability to assess fibrosis, which may lead to misclassification of disease severity^31^. The absence of histological confirmation further limits the interpretation of these findings in terms of true disease modification.

The biological plausibility of synbiotic effects in MASLD is supported by several mechanisms. Synbiotics may enhance intestinal barrier integrity, reduce endotoxin translocation, and modulate inflammatory signaling pathways along the gut–liver axis. Additionally, microbial production of short-chain fatty acids (SCFAs) may improve insulin sensitivity and regulate hepatic lipid metabolism^13^. Despite these plausible pathways, the present study did not include microbiome or metabolomic analyses; therefore, the proposed mechanisms remain speculative and primarily hypothesis-generating. Future studies incorporating 16S rRNA sequencing, metagenomics, or metabolite profiling (e.g., SCFAs, bile acids) would substantially strengthen the mechanistic interpretation of clinical findings^32^.

From a clinical perspective, synbiotics appear to be safe and well tolerated, with no reported adverse events. However, it is important to emphasize that lifestyle modification remains the cornerstone of pediatric MASLD management^16^. In the present study, no structured lifestyle intervention was implemented, and participants were not monitored for changes in diet or physical activity. Therefore, the findings should not be interpreted as evidence that synbiotics can replace lifestyle modification, but rather as a potential adjunctive strategy.

Several limitations of this study warrant careful consideration. First, the 8-week duration is relatively short for a chronic condition such as MASLD, and does not allow assessment of long-term efficacy or sustainability of observed improvements. Longitudinal studies with follow-up periods of 6–12 months are needed to determine whether these effects persist over time. Second, the lack of detailed data on dietary intake and physical activity represents a significant source of potential confounding, limiting causal inference. Third, the use of FOS in the placebo group introduces the possibility of biological activity in the control arm, which may have reduced the observed effect size. Fourth, the absence of liver biopsy precludes definitive assessment of disease severity and histological response, and reliance on ultrasound limits diagnostic precision. Fifth, strain-specific CFU data were not available, which restricts reproducibility and comparison with other studies. Finally, the relatively small sample size limits generalizability and statistical power for detecting smaller effect sizes.

## Conclusion

This randomized controlled trial demonstrates that short-term synbiotic supplementation is associated with improvements in liver enzyme activities, lipid profile, BMI, and ultrasonographic steatosis grade in children with MASLD. These findings support the potential role of synbiotics as an adjunctive therapeutic strategy; however, they should be interpreted within the context of important limitations, including short intervention duration, lack of lifestyle control, and absence of mechanistic data.

Future studies should incorporate longer follow-up periods, detailed assessment of diet and physical activity, and advanced microbiome and metabolomic analyses to better elucidate the mechanisms underlying these effects and to determine their long-term clinical relevance.

## Data Availability

Due to patient confidentiality, the study data are not publicly available. However, a de-identified version can be provided to the corresponding author upon reasonable request.
